# The art of observation: a qualitative analysis of medical students’ experiences

**DOI:** 10.1186/s12909-019-1671-2

**Published:** 2019-06-26

**Authors:** Bowen He, Smriti Prasad, Robin T. Higashi, Heather Woodworth Goff

**Affiliations:** 1 University of Texas Southwestern Medical Center, 5939 Harry Hines Blvd, Dallas, TX 75390-8570 USA; 2 Department of Dermatology, University of Texas Southwestern Medical Center, 5939 Harry Hines Blvd, Dallas, TX 75390-8570 USA; 3 Department of Population and Data Sciences, University of Texas Southwestern Medical Center, 5939 Harry Hines Blvd, Dallas, TX 75390-8570 USA

**Keywords:** Qualitative research, Curriculum, Burnout

## Abstract

**Background:**

Although the inclusion of arts in medical school curricula has garnered attention, little is known about the effect of arts-based interventions on the behaviors, attitudes, and technical skills of students. The Art of Observation is an optional elective at the University of Texas Southwestern Medical Center (UTSW) in collaboration with educators from the Dallas Museum of Art (DMA). We utilized a qualitative approach to describe in-depth how engaging with art influences the development of medical students’ observation skills and empathy.

**Methods:**

We analyzed evaluations from 65 medical students who completed the course between 2015 and 2017. Evaluations contained open-ended questions that asked students to reflect upon their experiences and describe their perceptions, thoughts, and feelings after guided museum visits. We used grounded theory to generate a thematic codebook, then employed axial coding to discover thematic relationships.

**Results:**

We report three main findings and several subthemes: (1) Enhanced observation skills: by engaging with art and completing relevant activities, students developed the ability to synthesize a compelling narrative in addition to learning technical skills; (2) Improved physician socialization: students reported enhanced self-awareness, increased tolerance of ambiguity, and development of a humanistic view of medicine, key components of physician socialization; and (3) Reduction in burnout symptoms: students reported an enhanced sense of well-being after each session, which mitigates the process of burnout.

**Conclusions:**

Fine arts can be used to teach technical skills, stimulate personal reflection, and prevent burnout. A meaningful engagement with the arts can play an important role in developing physicians who are observant, empathetic, and more well-rounded.

**Electronic supplementary material:**

The online version of this article (10.1186/s12909-019-1671-2) contains supplementary material, which is available to authorized users.

## Background

The inclusion of arts in medical school curricula has garnered much attention regarding its potential to improve observation skills, foster empathy, and promote reflection [1–5]. In order to justify including arts-based courses as an integral part of medical education, there is a need for studies that assess the effect of arts-based interventions on behaviors, attitudes, and technical skills of students. However, many educators have argued that judging the usefulness of the arts by technical standards fails to adequately capture the complex effects that art has on the process of growth and development of medical students [1].

The focus of arts-based courses has shifted away from developing mere technical observational and exam skills towards using arts to stimulate personal reflection and engage students in a holistic and humanistic way [6]. Engaging with art gives students an opportunity to broaden their perspectives, gain insight into both medical practice and themselves, and explore different ways of deriving meaning [7]. Arts-based courses have provided alternate means for students to develop professionalism and empathy, both of which are difficult-to-teach topics that are recognized as basic competencies in medical education [8]. This elective was created to encourage self-reflection, foster teamwork and teach skills relevant to clinical practice. In this paper we utilized a qualitative approach to provide a richer understanding of the ways in which our arts-based elective *The Art of Observation* influences the development of medical students’ observation skills and empathy.

## Methods

### Data collection

Evaluations were gathered from first, second and third-year medical students participating in *The Art of Observation* elective at the University of Texas Southwestern Medical School from 2015 to 2017. This course is comprised of 7 two-hour sessions, of which students must attend at least 5 sessions to receive transcript credit. Topics covered in various museum visits focus on topics including empathy, observation skills, compassion, and cultural concepts of health. The course format and objectives are available in an Additional file [see Additional file 1]. During these three years, a total of 92 students enrolled in the course and 65 students (71%) completed the course. Course evaluations contained open-ended questions that asked students to reflect upon their experiences and describe their perceptions, thoughts, and feelings after the sessions. Reflection prompts encouraged students to look for connections between the activities and the practice of medicine.

Students were informed that evaluations are an essential component of the elective and that these evaluations are used to improve future curriculum. Evaluations were de-identified prior to analysis.

### Data analysis

We used grounded theory [9] to guide our analysis of the evaluations and discover emerging themes. Two investigators (BH, HWG) independently read all evaluations line-by-line and used open coding [9] to generate preliminary themes and create a codebook, which was refined by consensus among all investigators. The resulting themes were discussed with a third investigator (RTH) experienced in qualitative methodology. We then employed axial coding to identify sub-themes and discover relationships between the major themes. We re-analyzed the data until we achieved theoretical saturation.

All elements of this study were approved by the medical school’s institutional review board.

## Results

We analyzed all evaluations written by medical students who completed the course (*n* = 65). We discovered three major themes and several subthemes from the data: [1] enhanced observation skills, [2] improved physician socialization through development of empathy, compassion, adoption of a non-judgmental perspective, and collaboration in complex problem-solving, and [3] reduction in burnout.

### Theme 1: enhanced observation skills

Nearly all students described an improvement in the skills necessary to accurately identify and analyze the key details in a visual work without jumping to conclusions. They learned to notice details that were initially overlooked and emphasized the importance of taking time to conduct a deep, thorough examination.

“I have learned how to appreciate, consider, and examine small details and variations and to not discredit them as unimportant.”

Students also developed a more focused and deliberate process for observation. They often recognized that their first impressions included bias and could only provide a limited understanding of the artwork.

“I feel that I had more insight into things that I may have missed when I initially look at an art object. I think this skill will apply to my medical practice because it will help me to be more observant about conditions a patient will present with and to perhaps not to take for granted certain visual findings that may appear at first insignificant.”

Students cultivated a more nuanced and deep understanding of artwork, a skill that can improve a physician’s perception of visual cues in a clinical setting.

“The way a patient walks into a room can tell you a lot about the patient’s mood, affect, energy level. Looking at art forces you to dissect and interpret on looks alone, so this has strengthened my ability to observe.”

Students recognized and characterized emotions in artworks and incorporated the context and surrounding cultural and historical information to formulate an impression from multiple sources of information. They integrated their own personal background and experiences to transform their impressions into a unique perspective.

“History adds depth. Patients, like the [art] works aren’t just this person/thing in the present. They have a past that adds to the complexities.”

One student concluded that close observation could reveal valuable information about an individual’s disease or emotional state and provide a useful toolkit when taking a patient history.

“I think it will really help us improve our patient history aspect of our practice where we can focus on not only what our patient has to say to us but also the non-verbal aspects such as body language [and] culture [ …]”.

### Theme 2: process of physician socialization

Students developed their personal and professional identities during the course, which is an important element of physician socialization, the process through which medical students develop the ethical values and professionalism that is expected of physicians [15].

Medicine is a collaborative field, and students commented that the team-based activities and adoption of a non-judgmental stance when evaluating a work of art and discussing various perspectives translated to the clinical environment. These comments portrayed course activities as valuable real-life experiences; students associated the analysis of a complex work of art with classmates with the collaborative skills that are needed in medical practice.

“Medicine, by its very nature, is a collaborative endeavor. By engaging in tasks that force us to work as a team we gain valuable experience communicating which will translate nicely into our roles as future physicians.”

“Everyone has different strengths, personalities, and experiences which translates to the juggling act and balance and practice of medicine. Different teams approached activities differently and it all depends on what works best for each specific team.”

A key component of physician socialization is the ability to communicate the complex and abstract topics that comprise the field of medicine. Students indicated that this course strengthened their ability to communicate abstract ideas, a skill that many students described as relevant to patient presentations in clinical practice. Students were often impressed with the insight of their classmates and commented that teamwork allowed the group to solve more difficult problems.

“The collaboration exercises in this course were good practice in communicating with each other to express abstract feelings and opinions.”

Another component of physician socialization is the tolerance of ambiguity. Students learned how to accept that there is sometimes no right or wrong answer, acknowledging that medicine, much like artwork, often has many grey-zones in which multiple choices may be correct. As one student succinctly summarized:

“[…] one thing can be interpreted a million different ways.”

### Another student elaborated

“Interpretation and analysis of art is such a personal and abstract thing. In medicine, we are taught to analyze concrete data and interpret facts, but I think that our approach to patients has to be more than that.”

One of the fundamental goals of physician socialization is to promote a humanistic view of medicine that advocates an unbiased and compassionate approach to others. Students desired to look beyond physical appearances and symptoms and instead strive to reconstruct the patient’s narrative. One student remarked:

“I understand that patients are similar to artwork in that each one tells a certain story.”

Another student reflected:

“Art is a wonderful vehicle to develop empathy and compassion and one I will continue to use.”

### Theme 3: reduction in burnout symptoms

Students viewed museum visits as a unique space that stimulated personal reflection and promoted a sense of enrichment and well-being. These sessions were often described as a temporary but necessary respite from the rigid medical school curriculum.

“[…] I often felt reinvigorated and refreshed after class, Going to the DMA for the first time and seeing all the folks gathered around for a night of art, refreshments, and jazz reminded me that life goes on outside of med school. All the text and slides we have to wade through is ultimately to help these people all around us […] [this] will help me become a more a more thoughtful, understanding physician.”

“This class was rejuvenating for me. I think it’s a major reason why I did not go insane this semester. Art is so healing, and I think all of us should make more time to experience it.”

## Discussion

This qualitative analysis of student course evaluations demonstrates that *The Art of Observation* elective positively impacted medical students in three overall ways: [1] It enhanced their observational skills, particularly in the development of a focused, deliberate process for analysis, as well as a nuanced and deep understanding of artwork and visual cues. [2] It contributed to developing positive physician socialization skills like teamwork, communication of complex thoughts, development of a humanistic view, and a tolerance of ambiguity. [3] It improved students’ overall wellness, which thereby contributes to preventing burnout.

The enhancement of observation skills from studying artwork has been described extensively in previous literature [1, 2, 4, 5, 10–18]. These studies have found that clinical diagnoses involve observation, description and interpretation of visual information, which are skills that can be garnered and enhanced by examining works of art [5]. One study described that through art, “one is forced to assess the information, to question the artist, […] the subject, […] and the viewer’s response, in order to gain understanding” [5], which improves both processing and communication skills. Another study found that observing arts allowed students to see beyond what was expected and help in development of complex pattern recognition [2]. Further studies concluded that the collaborative, critical thinking process that ensues from looking at art is parallel – and vital – to effective clinical practice [18]. In our study, we also found that students felt studying artwork helped them use a focused and deliberate process of observation to create a deep and nuanced understanding. Importantly, we noticed that the most poignant comments emphasized the ability to synthesize a narrative from the distinct and seemingly unrelated details in either a work of art or a clinical vignette, thus pointing to the possibility that the development of observation skills is not an independent outcome, but rather a component of physician socialization.

Physician socialization has been traditionally regarded in terms of teaching competencies that can be measured objectively [19]. Recently, educators have thought about physician socialization in terms of the *process* through which “humanistic competencies” [8] are achieved. The arts, inherently metaphorical and subjective, act as a medium through which educators can discuss ambiguous and complex topics and encourage medical students to integrate the combination of physical characteristics, emotions, and history that shape human perception. These unique properties allow art to enhance the personal growth of medical students and facilitate the development of a humanistic and empathetic professional identity [2, 20]. Arts-based sessions allow students to be aware of their feelings of uneasiness, nervousness, discomfort or anxiety [2] and hence, develop a tolerance for ambiguity. Literature supports that questioning assumptions is a key concept introduced in arts-based groups [2, 11, 14], and this allows for individuals to imagine experiences beyond their own, as well as recognize that uncertainty is an inherent part of medicine [18]. Furthermore, discussing these feelings in the context of art helps develop communication skills needed to articulate thoughts and craft a public narrative with others [15]. In our study, students reported enhanced self-awareness, increased tolerance of ambiguity, and development of a humanistic view of medicine, which supports this novel way of thinking about physician socialization.

Students also found that the small group discussions helped build teamwork and communication skills. Many students described the importance of teamwork in clinical practice. Although teamwork has become a major focus in healthcare and is a core competency in medical education, there is limited literature on the use of arts and humanities to teach teamwork and communication skills [21]. We found that small group discussions during *The Art of Observation* sessions allowed students to practice communicating their interpretations and allowed students to become aware of the perspectives of their peers. The process of learning and growth of students is enhanced when learners engage and process their thoughts in the context of a group [20]. We believe that the group discussion of fine works of art enhances self-reflection, a key component of physician socialization.

It is well-accepted that the arts can promote general well-being of physicians [22]. They are an outlet for personal enjoyment and creativity, especially in the midst of a rigorous medical education curriculum. Studies have found that extracurricular activities in the arts help promote student well-being [16], which was corroborated in narrative course evaluations. Art courses allow for students to reflect on their role as a physician and the challenges they face as they assume this role [10], and we believe this self-reflection is key to preventing physician burnout. Observing or practicing art forces one to slow down and take a step back from the hectic, and often stressful, life of practicing medicine.

*The Art of Observation* elective was designed to meet several objectives. The first was to teach students relevant clinical skills, including the ability to observe, interpret, and communicate visual information. The second was to encourage self-reflection and promote a humanistic understanding of medicine. The final objective was to provide opportunities for students to work in teams and gain exposure to diverse perspectives. The goal of this study was to understand how students translated the activities that they completed in the course to their medical practice. The student evaluations and qualitative analysis have allowed us to discover how our course has impacted students across the years.

### Limitations

In validation of the themes discovered through our analysis, comments submitted were thoroughly analyzed until thematic saturation was achieved to draw conclusions regarding the impact of this elective on student’s development as physicians-in-training. However, limitations still exist. Because the Art of Observation is an optional elective rather than required curriculum, participants form a self-selected group with a demonstrated interest in medical humanities. A small number of evaluations were incomplete, and some students who participated in the course did not submit an evaluation. Students’ comments represent impressions of how they changed throughout the course, but written assessments were performed upon course completion. Therefore, the reflections and opinions expressed in the evaluations received may not be representative of all students who enrolled in the course, and the data may not capture all the changes that occur through the process of the elective. Many students are also in their preclinical years of training and may not be able to make as many connections to clinical practice as students actively engaged in patient care.

## Conclusions

Medicine and art have several parallels. Both emphasize the importance of learning how to read gestures and expressions, how to interpret context, how to determine what is symbolically as well as literally important, how to be skeptical about initial assumptions, and finally, how to empathetically perceive emotional dimensions and narrative [2]. One of the major challenges in medical education is training students to become physicians who are not only competent, but also humanistic in their approach to patient care. This study revealed themes that support the role of the arts in developing physicians who are observant, empathetic, and better-rounded. Future study should attempt to combine quantitative and qualitative methods of assessing the growth and development of medical students through study of the arts. The durability of these positive transformations throughout the students’ medical training is yet unknown; therefore, we intend to reassess with a future project to better ascertain how the students’ experiences from this course transfer to behavioral changes.

## Additional file

Additional file 1: Art of Observation Course Description. (PDF 1240 kb)

## Data Availability

The data generated and analyzed during the current study are not publicly available to protect the privacy of the participants. The full raw data contains information that could compromise research participant privacy.

## Abbreviations

DMA: Dallas Museum of Art
UTSW: University of Texas Southwestern Medical Center
