# Supplementary material for: The art of observation: a qualitative analysis of medical students’ experiences
Source: BMC Med Educ. 2019 Jun 26;19:234. doi: 10.1186/s12909-019-1671-2 (PMC6595600; doi:10.1186/s12909-019-1671-2)
Supplement: Supplementary file 1 — Art of Observation Course Description. (PDF 1240 kb) [file 12909_2019_1671_MOESM1_ESM.pdf]

2017 Art of Observation

Course Directors:
Bonnie Pitman, Distinguished Scholar in Residence, Edith O'Donnell Institute of Art History, University of Texas at Dallas
Heather Wickless, MD, (faculty sponsor) Assistant Professor of Dermatology, University of Texas Southwestern Medical Center (UTSW)
Amanda Blake, Interim Director of Education, Dallas Museum of Art
Courtney Crothers, UTSW Art Curator

RATIONALE

The Art of Observation is a preclinical elective focusing on developing skills for clinical diagnosis through looking at works of art. Through experiences with artwork, students in the course will improve visual literacy skills, which is the ability to observe, analyze, interpret, and make meaning from information presented in the form of an image and relates to both examining patients as well as artwork. The course uses the power of art to promote the analysis and communication necessary in addressing ambiguity in the physical exam and patient interaction.

We discuss factors influencing what we see, and how we interpret visual information. Other topics include conservation, artists with disease, empathy, physician burnout and cultural influences, with their implications for medical practice. Participants will cultivate habits of close observation, inspection, and cognitive reflections to shape his or her early medical career. Students will learn to synthesize observations and one's own knowledge and experiences as well as an awareness of the collaborative thinking process of the group, a skill vital to successful clinical practice.

The class will engage students in discussions, drawing and writing exercises, lectures, and interactive experiences that will foster communication. This is not an art history class and students need no previous training in art to participate. The course meets in accordance with the schedule at the Dallas Museum of Art, Nasher Sculpture Center, The Warehouse, The Crow Collection and UTSW Medical Campus.

OBJECTIVES

- Expand students' abilities in observation, description, interpretation, and analysis of visual information.
- Gain an awareness and understanding of the conscious and unconscious factors that influence observation and interpretation of visual information along with the implications for decision making.
- Provide opportunities for students to work in teams, as we do in clinical settings; thereby listening, analyzing, and further developing one another's observations and hypotheses.
- Deepen an awareness of the arts to understand the human condition.

FORMAT

- 7 two-hour sessions THURSDAYS 5-7pm
- Small group discussions and lectures
- Drawing, writing, or other creative activity each week
- Minimum of 20 students, capped at 30
- Classes are held at the Dallas Museum of Art, Nasher Sculpture Garden, The Warehouse, The Crow Collection and UTSW Campus

COURSE STRUCTURE

Each class meets at the designated museum for that date. We will discuss the agenda for that date and work in large and small groups directly with works of art in the galleries to develop visual literacy skills through observation, description, analysis, collaborative interpretations, and presentations of their findings. Each session has a wrap-up discussion for students to provide feedback and discuss how the activity might apply to clinical practice. Sessions will incorporate drawing, photography and writing.

READINGS

1. Pitman, Bonnie. *The Dallas Museum of Art: A Guide to the Collection*; Dallas Museum of Art, 2011.
2. Elkins, James. *The Object Stares Back: On the Nature of Seeing.* Harvest, Harcourt Press, 1996
3. Naghshineh, Sheila, Janet P. Hafler, Alexa R. Miller, Maria A. Blanco, Stuart R. Lipsitz, Rachel P. Dubroff, Shahram Khoshbin, and Joel T. Katz. "Formal Art Observation Training Improves Medical Students' Visual Diagnostic Skills." *Journal of General Internal Medicine* 23.7 (2008): 991-97. Web.
4. Schaff, Pamela B., Suzanne Isken, and Robert M. Tager. "From Contemporary Art to Core Clinical Skills: Observation, Interpretation, and Meaning-Making in a Complex Environment." *Academic Medicine* 86.10 (2011): 1272-276. Web.

STUDENT EVALUATIONS

Students attending 6/7 sessions and completing a course evaluation will fulfill requirements for course transcript acknowledgment and a passing grade. Evaluations are used to improve future curriculum.

Shuttle Transportation to and From Southwestern campus will be provided
